# Supplementary material for: The Synergistic Effect of Piperlongumine and Sanguinarine on the Non-Small Lung Cancer
Source: Molecules. 2020 Jul 3;25(13):3045. doi: 10.3390/molecules25133045 (PMC7411589; doi:10.3390/molecules25133045)
Supplement: Supplementary file 1 [file molecules-25-03045-s001.pdf]

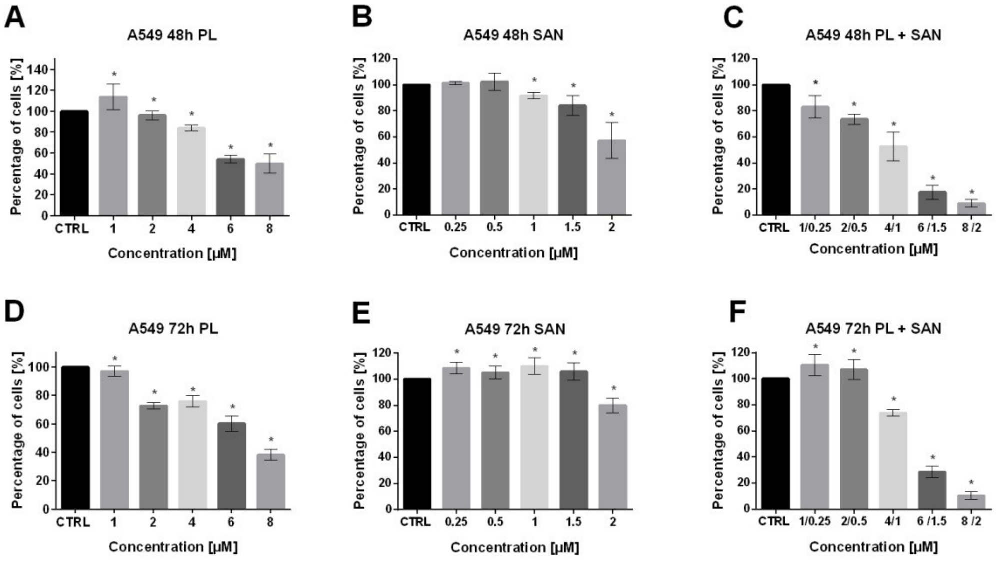

Figure S1. The cytotoxic effect of piperlongumine (PL) and sanguinarine (SAN) individually and in combined treatment on cell viability of A549 cells. The analysis was based on the results obtained from MTT assay. Cells were treated for 48 h and 72 h with PL at concentrations 1-8 µM (A, D), SAN at doses of 0.25, 0.5, 1, 1.5 and 2 µM (B,E) and their combination in ratio 4:1 (C, F). Data represent mean values ± SD obtained from 6 independent replicates (n=6). Statistically significant differences in comparison to untreated cells, where survival was estimated as 100%, were marked as '*' ($p<0.05$; Wilcoxon test).
